# MRI versus CT in the detection of brain lesions in patients with infective endocarditis before or after cardiac surgery

**DOI:** 10.1007/s00234-021-02810-y

**Published:** 2021-10-13

**Authors:** Paolo Vitali, Filippo Savoldi, Flavia Segati, Luca Melazzini, Moreno Zanardo, Maria Paola Fedeli, Adrienn Benedek, Giovanni Di Leo, Lorenzo Menicanti, Francesco Sardanelli

**Affiliations:** 1 Unit of Radiology, IRCCS Policlinico San Donato, Via Morandi 30, 20097 San Donato Milanese, Italy; 2 Postgraduate School in Radiodiagnostics, Università degli Studi di Milano, Milan, Italy; 3 Medicine and Surgery Medical School, Università degli Studi di Milano, Milan, Italy; 4 Department of Biomedical Sciences for Health, Università degli Studi di Milano, Via Mangiagalli 31, 20133 Milan, Italy; 5 Cardiac Surgery Department, IRCCS Policlinico San Donato, Via Morandi 30, 20097 San Donato Milanese, Italy

**Keywords:** Endocarditis, Subarachnoid hemorrhage, Abscess, Computed tomography, Magnetic resonance imaging

## Abstract

**Purpose:**

Imaging of brain involvement in infective endocarditis can drive the clinical management of this serious condition. MRI is very sensitive, but CT is more readily available. In this retrospective study, we compared the detection rates of CT and MRI.

**Methods:**

After Ethics Committee approval, we retrospectively reviewed a series of 20 patients (13 males, median age 64 years) who underwent both CT and MRI either before or after cardiac surgery for definite infective endocarditis. Plain CT and MRI were evaluated for acute ischemic lesions, both punctuate and large, intraparenchymal hemorrhages, cerebral microbleeds, subarachnoid hemorrhages, abscesses, microabscesses, and meningitis. Qualitative assessment and McNemar test were performed. The value of contrast-enhanced scans (MRI, *n* = 14; CT, *n* = 9) and cognitive status were also assessed.

**Results:**

A total of 166 lesions were identified on either technique: 137 (83%) on MRI only, 4 (2%) on CT only, and 25 (15%) on both techniques (p < 0.001). For these last 25 lesions, concordance on lesion type was only 16/25 (64%). MRI detected more microbleeds and ischemic lesions, while the 4 CT-only findings were false positives. Contrast-enhanced scans identified 68 enhancing lesions, mainly abscesses and microabscesses, and allowed a better characterization for 61/117 lesions (52%) with MRI, and for 11/81 (14%) with CT. Follow-up identified mild cognitive impairment in 6/13 and dementia in 3/13 patients.

**Conclusion:**

While CT rapidly excludes large hemorrhages in patients with infective endocarditis, MRI accurately distinguishes the whole spectrum of brain lesions, including small ischemic lesions, microbleeds, and microabscesses.

## Background

Infective endocarditis (IE) is defined as the infection of a native or prosthetic heart valve, the endocardial surface, or indwelling cardiac devices. IE is a serious disease that carries high mortality and morbidity as a consequence of primary heart involvement, among which valve regurgitation and heart failure, as well as of secondary end-organ damage due to the embolism from valve vegetations. Systemic embolism can be seen in left-sided endocarditis, when any organ might be affected, especially the brain. While neurological involvement occurs in about 15–30% of IE cases [1], up to 60% of patients show imaging signs of asymptomatic cerebral embolism [2, 3]. Neurological symptoms include focal deficit, encephalopathy, and seizures. Mycotic aneurysms result from the infection of the arterial wall and can lead to both symptomatic and asymptomatic bleeding. Albeit clinically silent, cerebral embolism, especially cortical microinfarcts [4], and microbleeds [5], may lead to long-term cognitive impairment.

The 2015 European Society of Cardiology Guidelines for the management of IE suggest that identification of brain involvement by imaging should be considered as a minor criterion (Duke criteria) for the diagnosis of IE, regardless of the presence of neurological symptoms [1]. Moreover, evidence of intracerebral hemorrhage is considered a contraindication to cardiac surgery, which should be postponed to up to 4 weeks. In addition, a ruptured mycotic aneurysm needs to be repaired before cardiac surgery, and at least two weeks should elapse between the two interventions [1]. These recommendations highlight the fundamental role of brain imaging in both diagnosis and management of IE.

Computed tomography (CT) is the most commonly requested examination, but magnetic resonance imaging (MRI) is also performed in more complicated clinical scenarios. However, there is no clear recommendation about which imaging modality should be performed in the setting of IE. As at our institution, we have real-world data on imaging practice in these patients, we aimed at comparing CT and MRI in detecting brain lesions in the setting of IE.

## Methods

### Patient selection

**Fig. 1:**
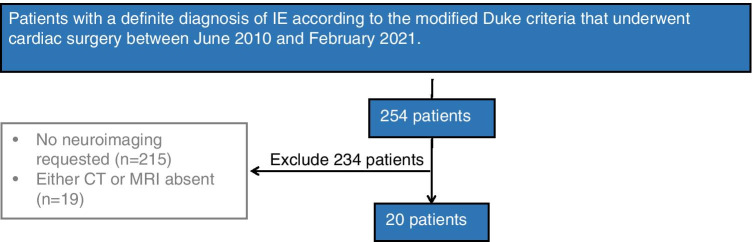
Flow chart of patient selection process

After Ethics Committee approval, we retrospectively reviewed all clinical records of patients with a definite diagnosis of IE according to the modified Duke criteria [1] who underwent cardiac surgery at IRCCS Policlinico San Donato, San Donato, Italy between June 2010 and February 2021. Of 254 such patients, 20 (8%) underwent both brain CT and MRI either before or after cardiac surgery; the remaining patients were excluded because either no brain involvement was suspected or no neuroimaging was requested (n = 215), or because either CT or MRI was not performed (n = 19) (Fig. 1).

Brain CT examinations were performed using multidetector scanners, either a 16-slice scanner (Somatom Emotion; Siemens Healthineers, Erlangen, Germany), a 64-slice scanner (Somatom Definition; Siemens Healthineers, Erlangen, Germany), or a 128-slice scanner (Go Top; Siemens Healthineers, Erlangen, Germany). When requested because of suspicion of cerebral abscesses, iodinated contrast material was administered (Iomeprolo, 400 mgI/mL, Bracco Imaging SpA, Italy, 1 mL/kg).

Brain MRI examinations were acquired using a 1.5-T unit (Symphony; Siemens Healthineers, Erlangen, Germany) equipped with 12-channel head coil. The imaging protocol included axial DWI (except for 1 patient), FLAIR, T2, T2* gradient echo (except for 4 patients), coronal FLAIR, sagittal T1. Detailed technical parameters are summarized in Table 1. When requested because of suspicion of cerebral abscesses, paramagnetic contrast material was administered (Gadoteridol, Bracco Imaging SpA, Italy, 0.1 mmol/kg).

**Table 1:** Technical parameters of MRI sequences

	TR (ms)	TE (ms)	TI (ms)	In-plane matrix	FOV (cm^2^)	Number of slices	Slice thickness (mm)
Axial DWI	9000	106		180 × 180	25 × 28	40	4
Axial FLAIR	6530	2127	108	320 × 272	25 × 28	40	4
Axial T2 TSE	6950	102		320 × 256	25 × 28	40	4
Axial T2* GRE	1260	25		512 × 384	25 × 28	40	4
Coronal FLAIR	5800	1980	106	256 × 212	25 × 28	40	4
Sagittal T1	633	8.7		320 × 320	25 × 28	40	4

Note: *DWI*, Diffusion-weighted imaging; *FLAIR*, Fluid-attenuated inversion recovery; *FOV*, Field of view; *GRE*, Gradient echo; *TE*, Time of echo; *TI*, Time of inversion; *TR*, Time of repetition; *TSE*, Turbo spin-echo

### Neuroradiological assessment

**Fig. 2:**
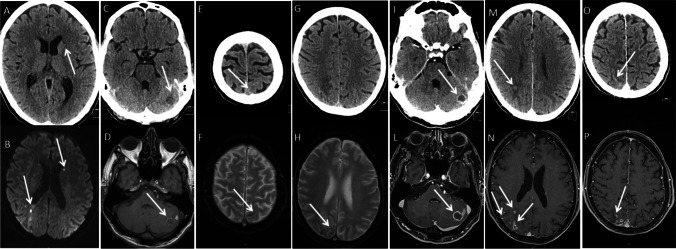
This figure summarizes the most common types of lesions identified in our study in a representative case who underwent both CT (top row) and MRI (bottom row), both plain (panels A-H) and enhanced (panels I-P). The following MRI sequences are shown: Diffusion-weighted image (DWI) (panel B), T2* weighted Gradient echo (GRE) (panels F, H), pre-contrast T1 weighted GRE (panel D) and post-contrast T1 weighted GRE (panels L-P). Arrows indicate punctuate ischemias (panel A, B), hemorrhages (panels C, D), subarachnoid hemorrhages (panels E, F), cerebral microbleeds (panel H, not clearly visible in G), abscesses (panels I, L), microabscesses (panels M, N), meningitis (panels O, P)

One radiology resident (Fi.S.) with four-year experience in neuroradiology, with the supervision of a senior neuroradiologist with 20-year experience (P.V.), blinded to the clinical status, reviewed all CT scans and, 10 days later, all MRI examinations. The following signs were evaluated on plain images: acute macroischemia (> 5 mm), acute punctuate ischemia (< 5 mm), intraparenchymal hemorrhages (> 5 mm), cerebral microbleeds (CMBs) (< 5 mm), subarachnoid hemorrhages (SAH), abscesses (> 5 mm), microabscesses (< 5 mm) and meningeal enhancement (meningitis) (Fig. 2). The 5-mm size cut-off for CMBs was chosen according to current standards [6]. If a single lesion had multiple components (e.g., large ischemic area with hemorrhagic foci), multiple attributes were assigned. Chronic ischemic changes of white matter were not recorded since more likely related to small vessels disease rather than to IE, the latter being more often embolic lesions at the cortico-subcortical junction in the watershed territories [3]. If contrast material was administered, the same reader reviewed all contrast-enhanced CT and MRI images in a separate session, recording any presence of enhancement and re-assigning the aforementioned attributes to each lesion. Any changes in the attributes between plain and enhanced images were then assessed to define the added value of contrast material.

### Clinical assessment

Because multiple brain lesions, even if asymptomatic at the time of imaging, are likely to affect the long-term cognitive status, all 20 patients were called through phone from 3 months to 10 years from the last imaging session. We performed a rough estimate of cognitive function by testing the reachable ones using the Italian telephone version of the Mini-Mental State Examination [7]. A potential association between cognitive status and brain lesions was then evaluated.

### Statistical analysis

Data are presented in terms of lesion detection rate as counts and percentages. Distributions were initially presented for plain examinations and the added value of contrast agent was given when available. Any difference between CT and MRI in terms of detection rate over the total number of lesions detected either at plain CT or MRI was ascertained using the McNemar test. Moreover, a potential correlation analysis between lesion burden and cognitive impairment was tentatively performed.

### STARD guidelines

The article follows the Standards for Reporting Diagnostic accuracy studies (STARD) guidelines [8]. Due to the nonquantitative nature of some variables, no estimates of diagnostic accuracy and their confidence intervals were provided.

## Results

### Clinical data

Thirteen patients were males and 7 females, with age at the time of the surgery ranging from 6 to 87 years, median of 64 years (25^th^ and 75^th^ percentile, 47–73 years).

Neurological signs were reported in the clinical records of 14 patients, both focal (diplopia, visual fields deficit, hemisyndrome with faciobrachiocrural motor deficit, facial weakness, vertigo, tongue paresthesia, dysarthria, dysesthesia) and general (impaired gait, somnolence, muscle weakness, neck stiffness, time–space disorientation, migraine with photophobia, psychomotor impairment). All patients underwent cardiac surgery to remove valve vegetations and replace or repair the cardiac valve(s) (aortic in 11 patients, mitral in 6, both in 3). All patients were treated with antibiotics.

### General imaging-related information

Table 2 lists the most relevant CT and MRI data for each patient. Patients underwent CT and MRI within a median of 3 days (range 1 − 18 days). In 5 cases, imaging was performed before surgery, in 15 cases after surgery. In two cases, CT and MRI were performed more than 3 months after surgery and no acute lesions were identified. No lesions at CT were demonstrated in 4 patients; in no case was MRI negative.

**Table 2:** Type of available scans, timing from surgery and types of lesions identified for each single patient

Patient #	Scans available				Time from surgery [days]		Types of lesions [number]															
	CT		MRI		CT	MRI	Large Ischemia (> 5 mm)		Punctuate ischemia (< 5 mm)		Intraparen-chimal hemorrhage		CMBs		SAH		Abscess (> 5 mm)		Micro-abscess (< 5 mm)		Meningitis	
	B	C	B	C			CT	MRI	CT	MRI	CT	MRI	CT	MRI	CT	MRI	CT	MRI	CT	MRI	CT	MRI
1	●	●	●	✘	8	11	1	1	0	0	0	0	0	0	0	0	0	0	0	0	0	0
2	●	✘	●	●	− 4	− 2	3	5	0	7	0	0	0	4	1	1	0	5	0	5	0	2
3	●	●	●	✘	− 15	− 17	1	0	0	0	0	1	0	21	0	0	1	1	0	0	0	0
4	●	✘	●	●	86	87	6	5	0	6	1	1	0	8	0	0	0	5	0	2	0	0
5	●	●	●	●	− 6	− 3	1	2	0	0	0	0	0	0	0	0	0	0	0	0	0	0
6	●	✘	●	●	50	52	0	0	0	0	0	0	0	0	0	0	0	0	0	0	0	1
7	●	✘	●	●	22	27	1	2	0	1	0	0	0	0	0	0	0	1	0	4	0	0
8	●	✘	●	✘	8	15	0	3	0	4	0	0	0	1	0	1	0	0	0	0	0	0
9	●	✘	●	●	3	8	1	0	0	0	0	0	0	1	0	1	0	0	0	0	0	0
10	●	●	●	●	3	6	0	0	0	0	0	0	0	0	0	1	0	0	1	1	0	0
11	●	●	●	●	6	8	0	0	0	0	0	0	0	0	1	1	0	0	0	0	0	0
12	●	✘	●	✘	95	99	0	0	0	0	0	0	0	5	0	0	0	0	0	0	0	0
13	●	✘	●	●	3	15	1	1	0	0	0	0	0	0	0	0	0	1	0	0	0	0
14	●	✘	●	✘	574	575	0	0	0	1	0	0	0	6	0	1	0	0	0	0	0	0
15	●	✘	●	✘	30	42	0	0	0	0	1	1	0	15	0	0	0	0	0	0	0	0
16	●	●	●	●	77	82	2	6	0	1	0	0	0	3	0	0	4	6	1	0	0	0
17	●	●	●	●	0	− 1	3	1	0	2	0	2	0	14	0	2	3	2	1	8	0	0
18	●	●	●	●	2	20	1	1	0	0	1	1	0	8	0	0	1	1	0	0	0	0
19	●	●	●	●	− 99	− 95	1	3	0	6	1	1	0	2	0	0	0	3	0	11	0	0
20	●	✘	●	●	− 4	− 3	2	4	0	1	3	3	2	10	0	0	0	4	0	2	0	0

Legend: *B*, baseline; *C*, contrast; *CMB*, cerebral microbleed; *CT*, computed tomography; *C* + , with contrast; *MRI*, magnetic resonance imaging; *SAH*, subarachnoid hemorrhage; ●, present; ✘, absent

Note: in patient #5 and #9 three and one lesions respectively were called acute at CT but were instead chronic at MRI; in patient #17 intraparenchimal hemorrhage was identified only at MRI since it was in a late stage, hypodense and blurrier at CT, where it was mislabeled as ischemia

### Plain CT and MRI

Table 3 summarizes the overall number of lesions identified on plain CT and MRI scans. Pooling the data of the two techniques, a total of 166 lesions were identified, most of them (*n* = 137; 83%) visible on MRI only; 25 (15%) were visible on both techniques, while only 4 (2%) were visible on CT only. The difference between the two detection rates was significant (*p* < 0.001).

**Table 3:** Contingency table with the overall number of lesions identified on plain CT and MRI scans in 20 patients before/after surgical treatment of infective endocarditis

**Imaging modality**		**MRI**		
	**Number of lesions**	**Absent**	**Present**	**Total**
**CT**	**Absent**	11^*^	137	148
	**Present**	4	25	29
	**Total**	15	162	

^*^ This refers lesions not visible at baseline CT and MRI, but identified after contrast administration

*CT*, computed tomography; *MRI*, magnetic resonance imaging

Table 4 provides the frequency of lesion components detected on plain CT and MRI: multiple components per single lesion were identified in 16 cases on MRI and in 7 on CT, for a total of 183 lesion components on MRI and 36 on CT. The two most common components were CMBs and ischemic lesions, both punctuate and large. Among the 25 lesions identified by both techniques, concordance on the lesion type was only partial (*n* = 16, 64%). The most common lesion components not detectable on CT but identified on MRI in plain conditions were CMBs (96/158, 61%), followed by punctuate ischemic lesions (29/158, 18%), larger ischemic lesions (19/158, 12%), SAH (6/158, 4%) intraparenchymal hemorrhages (4/158, 2%) and abscesses (4/158, 3%) (Table 4).

**Table 4:** Frequency of brain lesion components by subgroups in plain CT and MRI

All types of lesion components	CT [29 lesions]		MRI [162 lesions]		Lesions identified on MRI only [137 lesions]		Lesions identified on CT only [4 lesions]	
	N ^*^	%^**^	N ^*^	%^**^	N ^*^	%^**^	N ^*^	%^**^
Large ischemia (> 5 mm)	21	58	33	18	19	12	4^***^	100
Punctuate ischemia (≤ 5 mm)	1	3	30	16	29	18	0	0
Intraparenchimal hemorrhage	7	19	11	6	4	3	0	0
CMBs	2	6	97	53	96	61	0	0
SAH	2	6	8	4	6	4	0	0
Abscess (> 5 mm)	3	8	4	2	4	3	0	0
Microabscess (≤ 5 mm)	0	0	0	0%	0	0	0	0
Meningitis	0	0	0	0%	0	0	0	0
Total	36	100	183	100	158	100	4	100

^*^ Each lesion can have multiple components, so that the total number of components may exceed the number of lesions

^**^ This percentage is calculated on the overall number of components

^***^ These were false positives on CT: round hypodensities in the white matter that were considered recent ischemic lesions but were frankly old ischemic on MRI

Note: *CT*, computed tomography; *MRI*, magnetic resonance imaging; *CMBs*, cerebral microbleeds; *SAH*, subarachnoid hemorrhage

Only 4 lesions were identified on CT but not on MRI. They were likely to be false positives, as they appeared as ischemic lesions on MRI.

### Contrast-enhanced CT and MRI

Fourteen patients received contrast-enhanced MRI while 9 received contrast-enhanced CT; 7 had both contrast enhanced CT and MRI examinations.

Pooling the data from CT and MRI, a total of 68 enhancing lesions were detected: they were diagnosed as abscesses (28 on MRI, 9 on CT), microabscesses (34 and 3), meningitis (3 and 0), and subacute ischemia (1 and 1, respectively). Contrast-enhanced CT allowed the identification of 5 new lesions not visible on plain scans (2 microabscesses, 2 abscesses and 1 subacute ischemia), while contrast-enhanced MRI allowed the identification of 11 new lesions (9 microabscesses, 1 meningeal enhancement and 1 subacute ischemia).

In the subgroup of 7 patients who received both contrast-enhanced CT and MRI, we identified 34 enhancing lesions. Eleven lesions were identified on both techniques and there was complete concordance on the type of lesion between the two techniques: 7 (63%) were macro-abscesses, 3 (27%) were microbscesses, and 1 (10%) was a subacute ischemia. Twenty-three lesions were identified on MRI only: they were microbscesses (*n* = 18, 78%) and macro-abscesses (*n* = 5, 22%). No lesions were missed on MRI but identified on CT. Twenty-four lesions that were detected on plain examinations did not enhance after contrast administration.

In all 20 patients, in addition to the detection of new lesions that went undetected in plain examinations, the intravenous administration of contrast agent, when performed, allowed a better characterization of 61/117 lesions (52%) detected on MRI and of 11/81 lesions (14%) detected on CT. In particular, for MRI, the use of contrast agent allowed the definition of abscess in 24 cases considered as large ischemia in plain conditions; similarly, it allowed the definition of 12 microabscess in 5 cases of punctuate ischemia and 7 cases of CMBs. For CT, the use of contrast agent allowed the definition of abscess for 2 lesions initially considered as large ischemia and of microabscess for 1 lesion initially thought to be a punctuate ischemia.

### Cognitive assessment

Five of 20 patients were not reachable by phone while two had passed away. Thirteen patients completed the Italian telephone version of the Mini-Mental State Examination: 4 of them were cognitively intact, 6 had mild cognitive impairment and 3 were demented (2 of whom were reported by family members to be unable to engage a telephone conversation). No clear association between lesion type and cognitive impairment was found. Indeed, the 3 cognitively impaired patients presented on MRI a large ischemic lesion, a large ischemic lesion and CMBs, and only CMBs, respectively. On the other hand, 2 of the 4 cognitively intact patients had only 1 ischemic lesion, while the other 2 had multiple lesions, including large and small ischemic lesions, CMBs, microabscesess, and macroabscesses.

## Discussion

In this study, we compared the performance of CT and MRI in the detection of cerebral lesions in patients with IE. Considering plain MRI and CT scans, the former allowed a significantly superior detection rate than that of the latter: of 166 lesions identified on either technique, 137 (83%) were on MRI only, 25 (15%) on both techniques, while only 4 (2%) were visible on CT only (*p* < 0.001). Moreover, even when CT and MRI agreed on the presence of a lesion, the concordance on the type of lesion was only modest.

In line with previous experiences, the majority of lesions that were missed by plain CT and identified on plain MRI were CMBs [9, 10]. This is due to the inherent sensitivity of T2*-weighted sequences to detect even minor hemoglobin degradation products. In our series, the overall percentage of patients with CMBs detected using MRI was higher compared to those reported in previous studies [9, 11, 12]. This might be explained by different population demographics or by different severity of IE; moreover, as most MRI examinations were performed after surgery, CMBs could also belong, at least in part, to the spectrum of surgical sequelae. Our experience also confirms that CMBs are more common after cardiac surgery. In addition, also cerebral amyloid angiopathy is associated with CMBs that have the same distribution as the one following cardiac surgery, further complicating the picture [13]. Interestingly, it is worth noting that in our sample CT showed CMBs in 2 patients, while there is no literature data supporting the detectability of such lesions on CT [11, 12].

On the other hand, the detection of large hemorrhagic lesions can be made confidently both with baseline CT and MRI, as demonstrated by similar detection rate in our series. These findings are consistent with previous studies comparing CT and MRI accuracy to identify cerebral IE lesions [11, 12]. Interestingly, however, we detected a higher number of SAH with MRI compared to CT [12], most likely due to detection of tiny sulcal remnants of late subacute blood that is easily identified on T2*-weighted sequences on MRI but is no longer visible on CT scans at the time of the examination. Repeated subarachnoid bleeding, especially if associated with CMBs, might configure a condition of cortical superficial siderosis: endocarditis can be therefore considered one of its possible substrates.

We also found a much higher number of lesions classified as punctuate ischemia with plain MRI compared to plain CT. While indeed the identification of large ischemic lesions can be usually made confidently both with MRI and CT, small ischemic lesions are more difficult to identify on CT scans, in part due to the small size, in part due to the inherent ability of DWI sequences to better detect these lesions, even when millimetric [14]. Therefore, some smaller and deeply-located CT hypodensities may not be clearly identified or may be difficult to classify as old ischemic lesions versus recent ischemic lesions on CT, while this difference is typically apparent on MRI. In our study, this was the case of 4 lesions that were identified only on CT and not on MRI scans: these lesions, which were wrongly classified as recent ischemic lesion by both readers, were later demonstrated to be old lesions on MRI thanks to their characteristics on DWI. These 4 lesions were the only ones that were identified exclusively on CT, not on MRI scans.

The 2015 revision of the Duke criteria included the identification of recent embolic events or infectious aneurysms by imaging as a minor criterion for IE, regardless of the presence of symptoms [1]. Since silent embolic events are very frequent in these patients [2, 3, 15], the use of neuroimaging should be taken into consideration every time a suspect of IE is raised. Notably, detection of embolic lesions on MRI has been demonstrated to upgrade the diagnosis from non-definite to definite IE in up to 51% of patients [2].

In our patients, the intravenous administration of contrast agent, when performed, allowed the identification of additional lesions and, most importantly, allowed a better lesion characterization. Contrast enhancement was indeed crucial for definition of abscesses and microabscesses. Notably, however, in our sample, the contrast material administration allowed a better lesion characterization in 52% of lesions with MRI, but only in 14% with CT. So, MRI should be anyway considered superior to CT.

Similarly to punctuate ischemic lesions, microabscesses were also more commonly detected on contrast-enhanced MRI than on contrast-enhanced CT, thanks to a better ability of MRI to detect small lesions and subtle enhancements. Identification of microabscesses could be of major interest when evaluating the proper antibiotic regimen in IE patients [1] and can be considered as an evidence of embolic events, potentially adding one minor Duke’s criterion to non-definite diagnoses of IE.

We also observed meningeal enhancement consistent with focal meningitis in 3 patients on contrast-enhanced MRI, but none on contrast-enhanced CT. Both cerebral abscesses and meningitis have shown to be a clear sign of failure of antibiotic treatment, thus representing a major need for a surgical intervention that would control the infection and prevent further complications such as sepsis [1].

Neuroimaging is crucial for the clinical management of patients with IE: it can provide indications or contraindications to cardiac surgery and it can guide the timing for surgery [16]. The three main indications for valve repair or replacement are heart failure, uncontrolled infection and need to prevent embolic events; conversely, cerebral hemorrhage is a major contraindication [1]. CMBs, which were the most common type of lesion in our series and in many other reports [2, 3, 17], have been proposed as a predictor of impending intracranial hemorrhage. However, they are not likely to share the same pathophysiology as embolic lesions, rather they are considered a subacute inflammatory microvascular phenomenon which is intrinsic to IE [11, 12, 17]. CMBs are indeed not part of Duke’s minor criteria and not a contraindication to cardiac surgery in current guidelines [1, 3, 12]. Moreover, as already mentioned, both cerebral abscesses and meningitis have shown to be clear signs of failure of antibiotic treatment, thus representing a major need for a surgical intervention [1].

Imaging can also guide the timing of surgery, which can influence the outcome and should be chosen according to the severity of cerebral infarction [18]. The lowest risk of postoperative bleeding is reached with immediate surgery in case of small ischemic stroke, while surgery should be performed within 2 weeks in case of moderate-severe ischemia. This finding highlights the importance of detecting even minor ischemic lesions for anticipating surgery rather than procrastinating it. Finally, in case of cerebral hemorrhages, postponing valve replacement of at least 4 weeks allows for better clinical outcomes [18].

We also explored the long-term cognitive sequelae of IE using the Italian telephone version of the Mini-Mental State Examination [7]. Most patients presented with mild cognitive impairment. Only 4 of 13 patients available at follow-up were cognitively intact. These preliminary data are far from being comprehensive and might have multiple confounders, but highlight a potential impact of IE on long-term cognitive status and deserve further investigations.

### Study limitations

This study has some limitations, most of them due to its retrospective design. Firstly, all patients had CT and MRI scans, but not all of them were contrast-enhanced: therefore, our assessment of the added value of contrast material administration for each technique is limited. Secondly, since CT and MRI examinations were not performed simultaneously, MRI may have shown some additional lesions that had developed in the interval between the 2 exams, even though the time frame between the 2 examinations was only within a median of 3 days (range 1 − 18 days). Additionally, CT and MRI examinations were performed either before or after cardiac surgery, with post-surgical sequelae in the latter acting as a possible confounder. Furthermore, the evaluation of the cognitive status with a telephone interview is not an optimal method in lack of a baseline complete evaluation and might suffer from multiple confounders, including the prolonged delay of some evaluations. Therefore, our neuropsychological evaluation was only exploratory. Finally, the evaluation of cases performed by the resident and the senior neuroradiologist was not separate and therefore we did not provide any assessment of interrater reproducibility.

## Conclusions

This study showed that in patients with IE, MRI identifies more lesions compared to CT, especially CMBs, ischemic lesions, both punctuate and larger, and microabscesses. Moreover, the addition of contrast-enhanced MRI allows a better lesion characterization in about half of lesions. Imaging is crucial for the diagnosis and management of IE. Identification of abscesses is important for proper antibiotic choice and, together with identification of meningeal enhancement, can provide indication for cardiac surgery. MRI can have a clinical impact by contributing to upgrading the diagnosis of IE and by providing indications for the timing of cardiac surgery. Therefore, while CT might be performed to rapidly exclude large hemorrhages before surgery, MRI should be preferred to accurately evaluate the whole spectrum of brain lesions. Further studies are needed to better assess the role of asymptomatic brain lesions, especially CMBs, in guiding clinical decisions and to better delineate the long-term cognitive sequelae of IE.

## Data and materials availability

The datasets used and/or analyzed during the current study are available from the corresponding author on reasonable request.

## Abbreviations

CT: Computed tomography
CMBs: Cerebral microbleeds
DWI: Diffusion weighted imaging
IE: Infective endocarditis
MRI: Magnetic resonance imaging
SAH: Subarachnoid hemorrhage
STARD: Standards for reporting diagnostic accuracy studies
